# Harnessing Endogenous Peptide Compounds as Potential Therapeutics for Severe Influenza

**DOI:** 10.1093/infdis/jiad566

**Published:** 2023-12-07

**Authors:** Alison C West, Christopher M Harpur, Mélanie A Le Page, Maggie Lam, Christopher Hodges, Lauren K Ely, Andrew J Gearing, Michelle D Tate

**Affiliations:** Centre for Innate Immunity and Infectious Diseases, Hudson Institute of Medical Research; Department of Molecular and Translational Sciences, Monash University, Clayton; Centre for Innate Immunity and Infectious Diseases, Hudson Institute of Medical Research; Department of Molecular and Translational Sciences, Monash University, Clayton; Centre for Innate Immunity and Infectious Diseases, Hudson Institute of Medical Research; Department of Molecular and Translational Sciences, Monash University, Clayton; Centre for Innate Immunity and Infectious Diseases, Hudson Institute of Medical Research; Department of Molecular and Translational Sciences, Monash University, Clayton; Centre for Innate Immunity and Infectious Diseases, Hudson Institute of Medical Research; Department of Molecular and Translational Sciences, Monash University, Clayton; Lateral Pharma Pty Ltd, Melbourne, Australia; Lateral Pharma Pty Ltd, Melbourne, Australia; Centre for Innate Immunity and Infectious Diseases, Hudson Institute of Medical Research; Department of Molecular and Translational Sciences, Monash University, Clayton

**Keywords:** host-directed therapy, influenza virus, cytokine, pulmonary disease

## Abstract

**Background:**

Excessive pulmonary inflammation and damage are characteristic features of severe influenza virus infections. LAT8881 is a synthetic 16–amino acid cyclic peptide form of a naturally occurring C-terminal fragment of human growth hormone with therapeutic efficacy against influenza. Shorter linear peptides are typically easier to manufacture and formulate for delivery than larger cyclic peptides. A 6–amino acid linear peptide fragment of LAT8881, LAT9997, was investigated as a potential influenza therapy.

**Methods:**

LAT9997 was evaluated for its potential to limit disease in a preclinical mouse model of severe influenza infection.

**Results:**

Intranasal treatment of mice with either LAT8881 or LAT9997 from day 1 following influenza infection significantly improved survival outcomes. Initiating LAT9997 treatment at the onset of severe disease also significantly improved disease severity. Greater disease resistance in LAT9997-treated mice correlated with reduced lung immunopathology, damage markers, vascular leak, and epithelial cell death. Treatment reduced viral loads, cytokines, and neutrophil infiltration in the airways yet maintained protective alveolar macrophages in a dose-dependent manner. Sequential trimming of N- and C-terminal amino acids from LAT9997 revealed a structure-activity relationship.

**Conclusions:**

These findings provide preclinical evidence that therapeutic LAT9997 treatment limits viral burden and characteristic features of severe influenza, including hyperinflammation and lung damage.

**Summary:**

Excessive pulmonary inflammation and damage are characteristic features of severe influenza virus infections. LAT9997 is a linear peptide fragment derived from human growth hormone. This study provides preclinical evidence that therapeutic LAT9997 treatment limits viral burden, hyperinflammation, and lung damage.

Severe influenza A virus (IAV) infections in humans are associated with hyperinflammation, leading to lung injury and the development of untreatable acute respiratory distress syndrome [1, 2]. Current therapeutic strategies for severe IAV largely focus on antivirals, which directly target essential viral proteins. However, the use of antiviral drugs such as oseltamivir is not associated with reduced mortality [3], potentially due to their limited efficacy if administered after the first 2 days of symptomatic infection, as well as the emergence of drug-resistant IAVs [4, 5]. Host-directed therapies that limit hyperinflammation and lung damage and offer reduced risk of drug resistance are urgently needed to increase our pandemic preparedness.

Growth hormone (GH) is a 4α-helical cytokine family member that regulates several biological processes via its cognate receptor [6, 7]. GH is known to be proteolytically processed in vivo at sites of tissue damage or pathology, resulting in new active peptides. At least 2 major biologically active fragments of GH have been identified: a large fragment comprising 3 N-terminal α-helices, which has antiangiogenic properties, and a smaller C-terminal fragment containing a disulphide-constrained loop [8, 9].

LAT8881 is a synthetic form of the C-terminal fragment of human GH, with the sequence YLRIVQCRSVEGSCGF, including 2 disulphide-linked cysteines [10, 11]. LAT8881 does not act via the GH receptor [12]. We recently determined that LAT8881 can interact with the lanthionine synthetase C-like protein (LANCL) family, which comprises peptide-modifying enzymes that support cell survival in the face of oxidative and chemical stress [13–15]. Consistent with this, LAT8881 treatment of cells in vitro enhanced cell viability, particularly in the presence of cytotoxic stress, which was countered by small interfering RNA inhibition of LANCL1 and LANCL2 [13]. Critically, daily intranasal treatment of mice with LAT8881 or its 10–amino acid cyclic metabolite, LAT9991F, following IAV infection improved survival, limited viral replication, reduced local inflammation, and curtailed tissue damage [13].

LAT8881 is a cyclic peptide that requires a multistep manufacturing process and presents difficulties in solubilization. A linear and smaller compound derived from LAT8881 would have a more favorable manufacturing profile, including reduced cost of goods. Therefore, we investigated the in vivo activity of the 6–amino acid linear peptide LAT9997, which encompasses the peptide sequence within the 2 disulphide-linked cysteines of LAT8881 (sequence RSVEGS). Intranasal administration of LAT9997 following IAV infection improved survival outcomes, which correlated with reduced viral burden, lung injury, and vascular leak. Moreover, LAT9997 treatment was associated with reduced levels of key proinflammatory cytokines and neutrophil accumulation in the airways. Numbers of alveolar macrophages (AMs)—key sentinels of the airways that are reduced following IAV infection—were maintained by LAT9997 treatment. Last, sequential trimming of amino acids from the termini of LAT9997 reduced therapeutic protection.

## METHODS

### Compound Generation

LAT8881 is a 16–amino acid synthetic form of the C-terminal fragment of human GH (H-YLRIVQCRSVEGSCGF-OH), which contains an additional N-terminal tyrosine residue and 2 cysteine residues linked by a disulphide bond. LAT9997 is a 6–amino acid synthetic linear peptide (RSVEGS). LAT9997scr (SGRVSE) is a scrambled control peptide. Additional peptides used: LAT9997-n1 (SVEGS), LAT9997-n2 (VEGS), LAT9997-c1 (RSVEG), LAT9997-c2 (RSVE), and LAT9997-c3 (RSV). All peptides were synthesized by Auspep Pty Ltd or GenScript Biotech Pty Ltd.

### Influenza Virus

The IAV strain HKx31 (H3N2) was grown in 10-day embryonated chicken eggs and titrated on Madin-Darby canine kidney cells.

### Influenza Virus Infection of Mice

Male and female C57BL/6J mice (6–8 weeks old) were maintained in the specific pathogen–free animal research facility (physical containment level 2) at the Monash Medical Centre. All experimental procedures were approved by the Hudson Animal Ethics Committee. Mice were intranasally inoculated with 10^4^ plaque-forming units (pfu) of HKx31 [13, 16]. Mice were therapeutically treated with peptides (5, 10, or 20 mg·kg^−1^) in 25 µL of phosphate-buffered saline (PBS) via the intranasal route. Control mice received PBS or LAT9997scr. Mice were weighed daily, assessed for clinical signs of disease, and humanely euthanized, as described in the supplementary material.

Mice were sacrificed via intraperitoneal injection of sodium pentobarbital. Bronchoalveolar lavage (BAL) was then immediately performed postmortem by flushing the lungs 3 times with 1 mL of PBS. Titers of infectious virus in lung tissues were determined by plaque assay on Madin-Darby canine kidney cells.

### Quantification of Cytokines in Mouse BAL Fluid and Sera

Levels of IL-6, MCP-1/CCL2, IFNγ, IL-10, IL-12p70, and TNF proteins were determined by cytokine bead array (mouse inflammation kit; BD Biosciences). Levels of keratinocyte-derived chemokine (KC)/CXCL1 were determined by a cytokine bead array flex kit (BD Biosciences).

### Flow Cytometry on BAL and Blood Cells

Leukocytes in BAL and blood were enumerated by flow cytometry as described in the supplementary material.

### Assessment of Lung Damage and Immunopathology

BAL fluid was assessed for levels of lactate dehydrogenase (LDH), adenosine triphosphate (ATP), S100 calcium-binding protein A10 (S100A10), and total protein levels as described in the supplementary material. Histologic examination of hematoxylin and eosin–stained lung tissue sections and TUNEL assay (terminal deoxynucleotidyl transferase-mediated dUDP nick-end labeling) were performed as described in the supplementary material.

### Data and Statistical Analysis

Data were tested for normality and analyzed by GraphPad Prism 9 software. Full details of statistical analysis and data availability can be found in the supplementary material.

## RESULTS

### Intranasal Treatment With LAT8881 or LAT9997 Promotes Resistance to Severe IAV Infection

**Figure 1.**
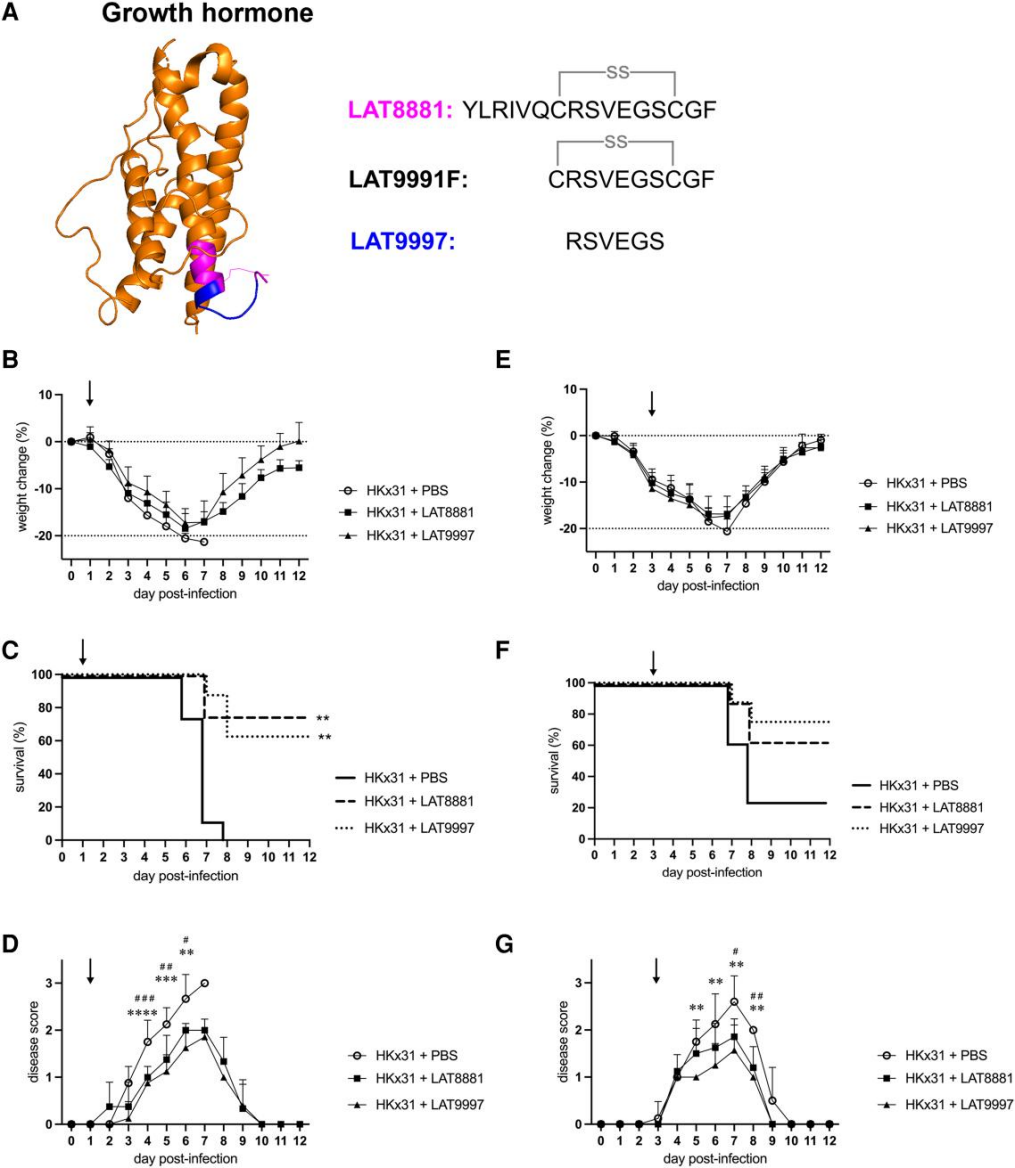
LAT8881 and LAT9997 improve resistance to influenza A virus infection in vivo. *A*, Schematic of human growth hormone structure (orange; http://www.rcsb.org/structure/1HGU). The synthetic compound LAT8881 is a cyclized peptide containing a cysteine-cysteine–bonded loop (SS) and is derived from the C-terminal region of growth hormone (magenta). LAT9991F is a cyclized peptide and a metabolite of LAT8881. The linear peptide LAT9997 consists of the 6 amino acids within the cysteine-cysteine–bonded region of LAT8881 (blue). Groups of male C57BL/6 mice (n = 8) received daily intranasal treatment with 20 mg·kg^−1^ of LAT8881, 20 mg·kg^−1^ of LAT9997, or PBS (vehicle control) from 1 day postinfection (*B–D*) or 3 days postinfection (*E–G*) with 10^4^ plaque-forming units of HKx31 influenza A virus. The downward arrow indicates the day that treatment was initiated. *B*, *E*, Mouse weight was recorded daily, and results are expressed as the mean ± SD percentage weight change. *C*, *F*, Survival curves. **P < .01: Mantel-Cox log-rank test. *D*, *G*, Each mouse was assigned a score of clinical disease daily (scale, 0–3; see Methods), and results are expressed as mean ± SD disease score. **P < .01, ***P < .001, ****P < .0001: PBS vs LAT9997. ^#^*P* < .05, ^##^*P* < .01, ^###^*P* < .001: PBS vs LAT8881. Two-way analysis of variance with Dunnett multiple-comparisons test. PBS, phosphate-buffered saline.

The development of new, safe, and effective host-directed therapies for severe IAV infections is urgently needed. We previously showed that therapeutic treatment of mice with LAT8881, as derived from the C-terminus of GH, or its metabolite LAT9991F improves survival outcomes and limits influenza disease severity [13]. LAT8881 and LAT9991F are 16– and 10–amino acid peptides, respectively, with both cyclized through a cysteine-cysteine disulphide bond (Figure 1*A*), which makes manufacture complex and costly. We therefore generated the linear peptide LAT9997, consisting of the 6 amino acids, from within the LAT8881 cysteine-cysteine–bonded loop. To investigate the in vivo therapeutic effects of LAT9997, mice were infected intranasally with 10^4^ pfu of HKx31 (H3N2) IAV [13]. Mice subsequently received daily intranasal treatments with 20 mg·kg^−1^ of LAT8881 or LAT9997 initiated at 1 (Figure 1*B–D*) or 3 (Figure 1*E–G*) days postinfection (dpi). Mice were euthanized upon losing 20% or more of their initial body weight or displaying a severe clinical disease score of 3 (supplementary methods).

Treatment with either 20 mg·kg^−1^ of LAT8881 or LAT9997 from 1 dpi via the intranasal route reduced weight loss, resulting in a significantly higher survival rate as compared with mice that received PBS vehicle (Figure 1*B* and 1*C*). Disease symptoms, including reduced mobility and rapid breathing, were also less severe over the course of infection in mice treated with LAT8881 and LAT9997 (Figure 1*D*, Supplementary Figure 1*A*). Initiation of LAT9997 treatment at 3 dpi with LAT8881 or LAT9997 slowed weight loss and reduced clinical signs of disease, resulting in improved survival (*P* = .057; Figure 1*E–G*, Supplementary Figure 1*B*). These data suggest that therapeutic treatment with the cyclized peptide LAT8881 or the shorter linear peptide LAT9997 limits disease symptoms when treatment is commenced early or at the peak of infection.

### LAT9997 Reduces Severe Pulmonary Pathology During IAV Infection

**Figure 2.**
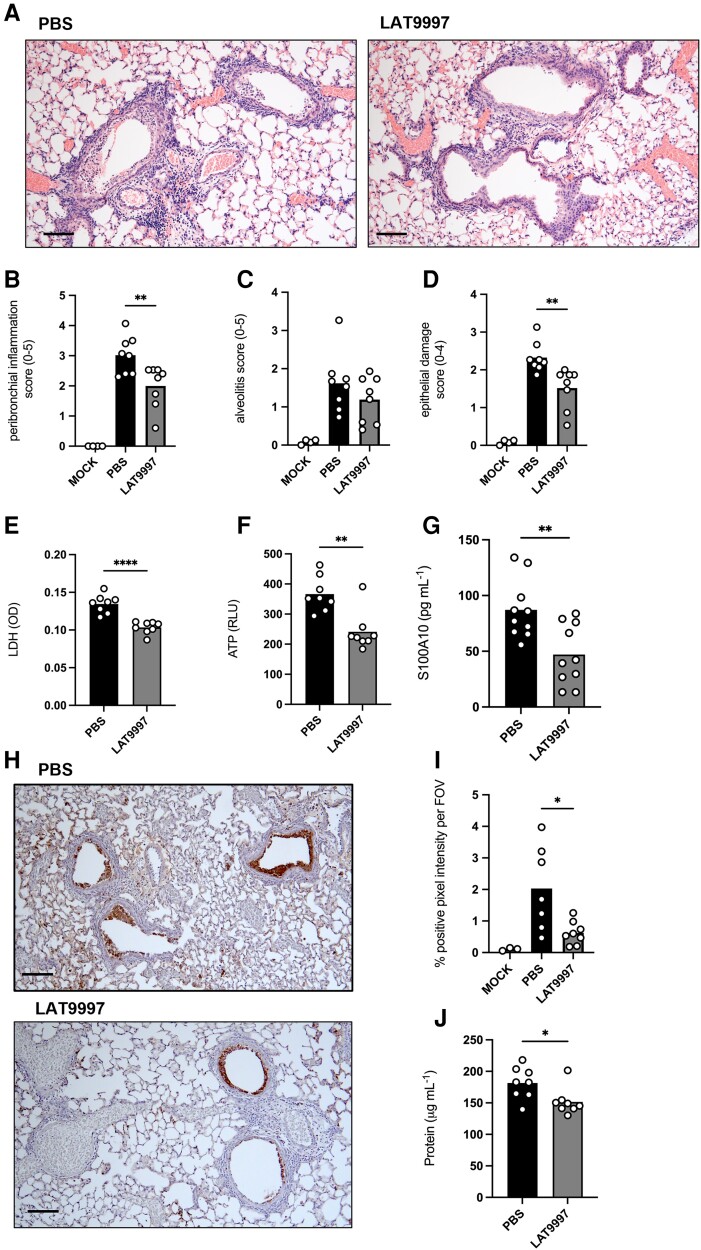
LAT9997 treatment of severe influenza A virus (IAV) infection reduces pulmonary immunopathology. Groups of male C57BL/6 mice were infected with 10^4^ plaque-forming units of HKx31 IAV. Mice received daily intranasal treatment with 20 mg·kg^−1^ of LAT9997 from 1 day postinfection, except for mice shown in panel *G*, which received 10 mg·kg^−1^ of LAT9997. Mock- and IAV-infected control mice received PBS alone. *A*, Histologic analysis of hematoxylin and eosin–stained lung tissue sections, with representative images at 10× magnification (scale bar, 100 μm). Lung sections were randomized and scored blindly by 3 readers for (*B*) peribronchial inflammation (scale, 0–5), (*C*) alveolitis (scale, 0–5), and (*D*) epithelial damage (scale 0–4), as described in the Methods section. Data are presented as the mean, with each data point representing an individual animal (n = 4–8). **P < .01: only PBS vs LAT9997 is shown. One-way analysis of variance with Dunnett multiple-comparisons test. Levels of (*E*) lactate dehydrogenase (LDH), (*F*) adenosine triphosphate (ATP), and (*G*) S100 calcium-binding protein A10 (S100A10) in bronchoalveolar lavage fluid were determined by colorimetric assay (optical density [OD]), luminescent assay (raw luminescence units [RLU]), or enzyme-linked immunosorbent assay. Data are presented as the mean, with each data point representing an individual animal (n = 8). **P < .01, ****P < .0001: Student *t* test. *H*, TUNEL assay labeling of cell death in lung tissue sections. Representative images at 10× magnification (scale bar, 100 μm). *I*, Quantification of TUNEL staining determined with ImageJ software as described in the Methods section. Data are presented as the mean percentage positive pixel intensity per field of view (FOV), with each data point representing an individual animal (n = 3–8). *P < .05: only PBS vs LAT9997 is shown. One-way analysis of variance with Dunnett multiple-comparisons test. *J*, Levels of protein in bronchoalveolar lavage fluid determined by colorimetric assay. Data are presented as the mean, with each data point representing an individual animal (n = 8). *P < .05: Student *t* test.

We hypothesized that a reduction in lung pathology was contributing to the reduced disease severity and improved survival upon 20 mg·kg^−1^ of LAT9997 treatment (Figure 1). Histopathologic analysis of hematoxylin and eosin–stained lung tissue sections at 3 dpi (Figure 2*A*) indicated that LAT9997 treatment from 1 dpi significantly diminished peribronchial inflammation with a more moderate reduction in alveolitis (Figure 2*B* and 2*C*). Additionally, LAT9997 treatment significantly reduced epithelial damage (Figure 2*D*). We also examined lung tissue sections from mice that were treated from 3 dpi with 20 mg·kg^−1^ of LAT9997 and had recovered from infection (euthanized at 12 dpi following return to starting weight; Figure 1*E*). Representative images are shown in Supplementary Figure 2. Intriguingly, resolution of peribronchiolar inflammation and restoration of the normal ciliated epithelium in the airways were observed in mice treated with LAT9997, while mice that received PBS displayed unresolved peribronchial inflammation and epithelial damage.

Extracellular LDH and ATP are released by dead or dying cells, making them indicators of tissue damage or damage-associated molecular patterns (DAMPs). Consistent with the results from the histopathologic analysis (Figure 2), 20 mg·kg^−1^ of LAT9997 treatment from 1 dpi significantly reduced LDH and ATP levels in BAL fluid (Figure 2*E* and 2*F*). S100A10 was also significantly reduced with 10 mg·kg^−1^ of LAT9997 (Figure 2*G*). To examine cell death, TUNEL labeling of lung tissue sections was undertaken (Figure 2*H*). LAT9997 at 20 mg·kg^−1^ significantly reduced cell death, particularly in epithelial cells, as compared with mice that received PBS (Figure 2*I*).

Inflammation and virus-induced lung damage can result in vascular leakage, which is characterized by the accumulation of protein-rich fluid in the airways [1]. BAL fluid protein levels are an indicator of vascular leak [17, 18]. Treatment with 20 mg·kg^−1^ of LAT9997 significantly reduced total protein levels in BAL fluids (Figure 2*J*). Together, these data demonstrate that LAT9997 treatment limits pulmonary tissue damage, including vascular leak and epithelial cell death.

### Dose-Dependent Modulation of Virus Dissemination and the Immune Response to IAV Infection by LAT9997

**Figure 3.**
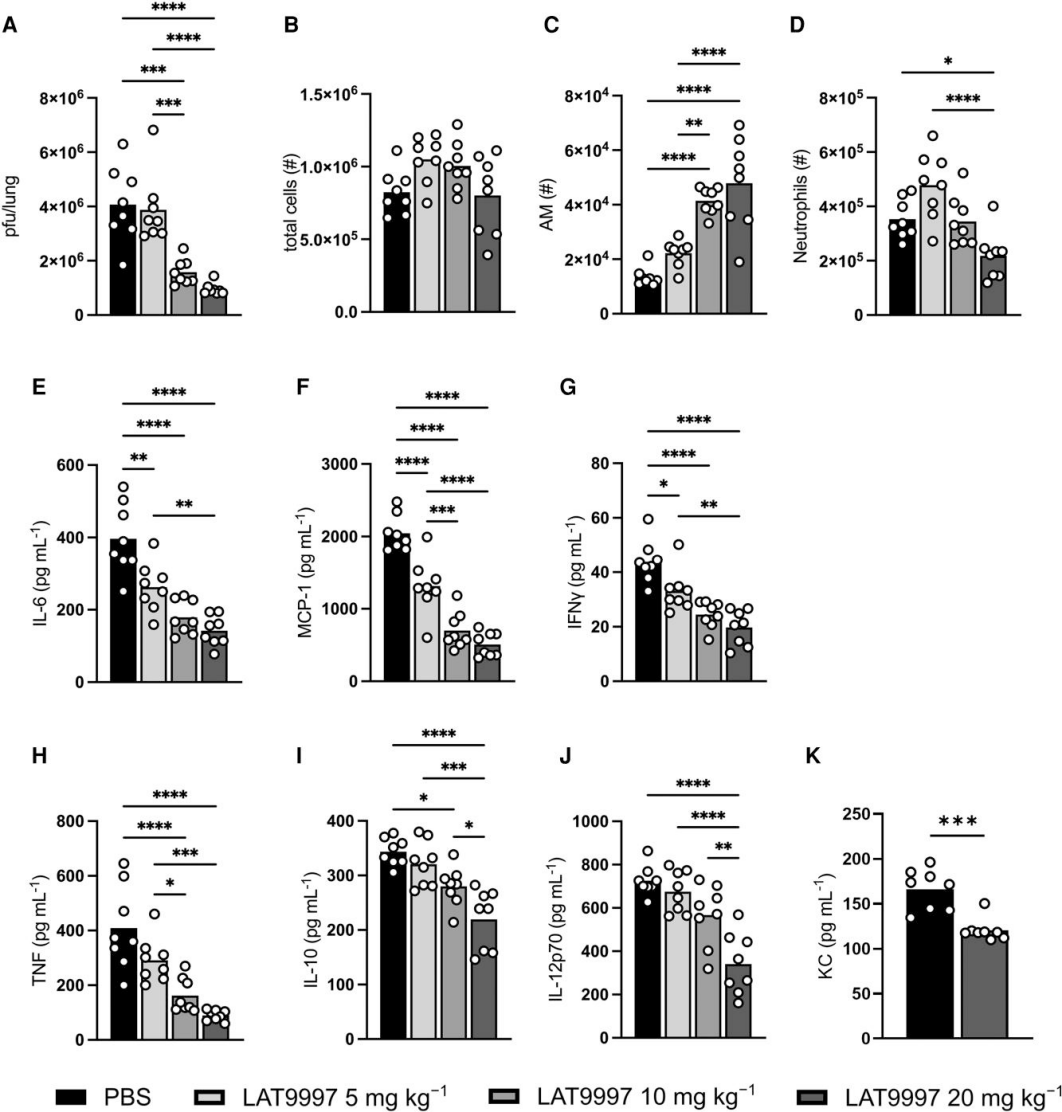
LAT9997 treatment limits the severity of influenza A virus (IAV) infection in a dose-dependent manner. Groups of male C57BL/6 mice received daily intranasal treatment with 5, 10, or 20 mg·kg^−1^ of LAT9997 from 1 day postinfection with 10^4^ plaque-forming units of HKx31 IAV. IAV-infected control mice received PBS alone. Bronchoalveolar lavage (BAL) fluid and lung tissues were collected at 3 days postinfection. *A*, Lung viral loads (plaque-forming units/lung) were measured by a standard plaque assay. *B–D*, Numbers of total cells, alveolar macrophages (AM), and neutrophils in the BAL fluid as determined by flow cytometry. *E–K*, BAL fluid concentrations of IL-6, MCP-1/CCL2, IFNγ, TNF, IL-10, IL-12p70, and KC/CXCL1 were determined by cytokine bead array. Data are presented as the mean from a single experiment, with each data point representing an individual animal (n = 8). *P < .05, **P < .01, ***P < .001, ****P < .0001. One-way analysis of variance with Tukey multiple-comparisons test. PBS, phosphate-buffered saline.

Severe IAV infections are characterized by immunopathology and tissue damage that result from excessive cellular inflammation and cytokine production at the site of infection and systemically [19, 20]. Therefore, we examined infectious viral loads in lung tissue as well as immune cells and inflammatory cytokines in BAL fluid and blood at 3 dpi (Figure 3, Supplementary Figure 3). LAT9997 was administered at increasing doses (5, 10, or 20 mg·kg^−1^) intranasally each day from 1 dpi. An additional IAV-infected control cohort received PBS.

Lung infectious viral burden (pfu/lung) was significantly reduced (>61%) by 10 or 20 mg·kg^−1^ of LAT9997 treatment (Figure 3*A*). We next enumerated total and immune cell subsets within the BAL at 3 dpi using flow cytometry [13]. All IAV-infected mice had comparable total airway cellularity (Figure 3*B*), as well as similar numbers of T cells, inflammatory macrophages (IMs), and dendritic cells (Supplementary Figure 3*A–C*). Airway natural killer cell number was similar among all IAV-infected mice, with a slight increase following 10 but not 20 mg·kg^−1^ of LAT9997 treatment (Supplementary Figure 3*D*). Resident AMs are susceptible to HKx31 IAV infection [18, 21]. AMs play an important protective role in limiting viral loads [18, 22] and susceptibility to secondary bacterial infection [23]. We previously showed that administration of 20 mg·kg^−1^ of LAT8881 resulted in greater numbers of AMs [13]. Here, LAT9997 treatment resulted in increased numbers of AMs at all doses administered, which was more pronounced with doses of 10 and 20 mg·kg^−1^ (Figure 3*C*). Neutrophils are crucial for protection against IAV [24]; however, they can also contribute to the development of disproportionate hyperinflammation and immunopathology [25]. Therefore, it is noteworthy that 20 mg·kg^−1^ of LAT9997 treatment significantly reduced neutrophil numbers in the airways (Figure 3*D*), in contrast to our previous results with LAT8881 [13].

LAT9997 treatment at all doses significantly reduced levels of IL-6, CCL2/MCP-1, and IFNγ in the BAL fluid when compared with mice treated with vehicle alone (Figure 3*E–G*). Additionally, TNF and IL-10 levels were significantly reduced in mice that received 10 and 20 mg·kg^−1^ of LAT9997 (Figure 3*H* and 3*I*), with IL-12p70 levels significantly reduced by 20 mg·kg^−1^ of LAT9997 (Figure 3*J*). KC/CXCL1 is a neutrophil chemokine and murine homologue of IL-8/CXCL8, produced by IAV-infected epithelial cells and AMs [21]. In line with the reduction in neutrophil numbers in Figure 3*D*, KC levels were significantly reduced following 20 mg·kg^−1^ of LAT9997 treatment (Figure 3*K*). Systemic levels of all assessed cytokines were largely unchanged by LAT9997 treatment (Supplementary Figure 3*E*–*J*).

While circulating leukocyte numbers at 3 dpi were comparable across all experimental cohorts (Supplementary Figure 4*A*), further immune cell phenotyping by flow cytometry revealed significantly elevated numbers of neutrophils and Ly6C^+^ major histocompatibility complex class II^+^ monocytes in IAV-infected mice as compared with mock-infected animals; yet, no significant differences were seen between the PBS and LAT9997 treatment groups (Supplementary Figure 4*B* and 4*C*). Severe IAV infections are commonly associated with lymphopenia [17, 18, 26]. Indeed, IAV infection was associated with reduced total blood lymphocytes in our model (Supplementary Figure 4*D*). IAV infection resulted in B- and T-cell lymphopenia, with natural killer cell numbers remaining essentially unchanged (Supplementary Figure 4*E–G*). Mice treated with 20 mg·kg^−1^ of LAT9997 had more moderate lymphopenia and possessed significantly greater numbers of circulating B cells, CD4^+^ T cells, CD8^+^ T cells, and double-negative T cells (Supplementary Figure 4*D*, *F–J*). Blood neutrophil-to-lymphocyte ratio (NLR) is typically elevated upon IAV infection and is used as an early prognostic indicator of susceptibility [27, 28]. Congruently, PBS-treated IAV-infected mice displayed a significantly increased NLR in comparison with mock-infected mice, while LAT9997 treatment resulted in a significantly less marked increase in blood NLR (Supplementary Figure 4*K*).

Collectively, these data suggest that local administration of LAT9997 increases influenza disease resistance through a dose-dependent decrease in lung damage, vascular leak, airway inflammation (proinflammatory cytokine and neutrophil infiltration), and lung viral burden. LAT9997 treatment also led to a greater retention of resident AMs—the primary immune sentinels of the airways that are crucial for restoring lung homeostasis. Finally, LAT9997 treatment reduced blood lymphopenia and NLR, prognostic markers of poor IAV outcomes.

### Identification of the Active Motif Within LAT9997

**Figure 4.**
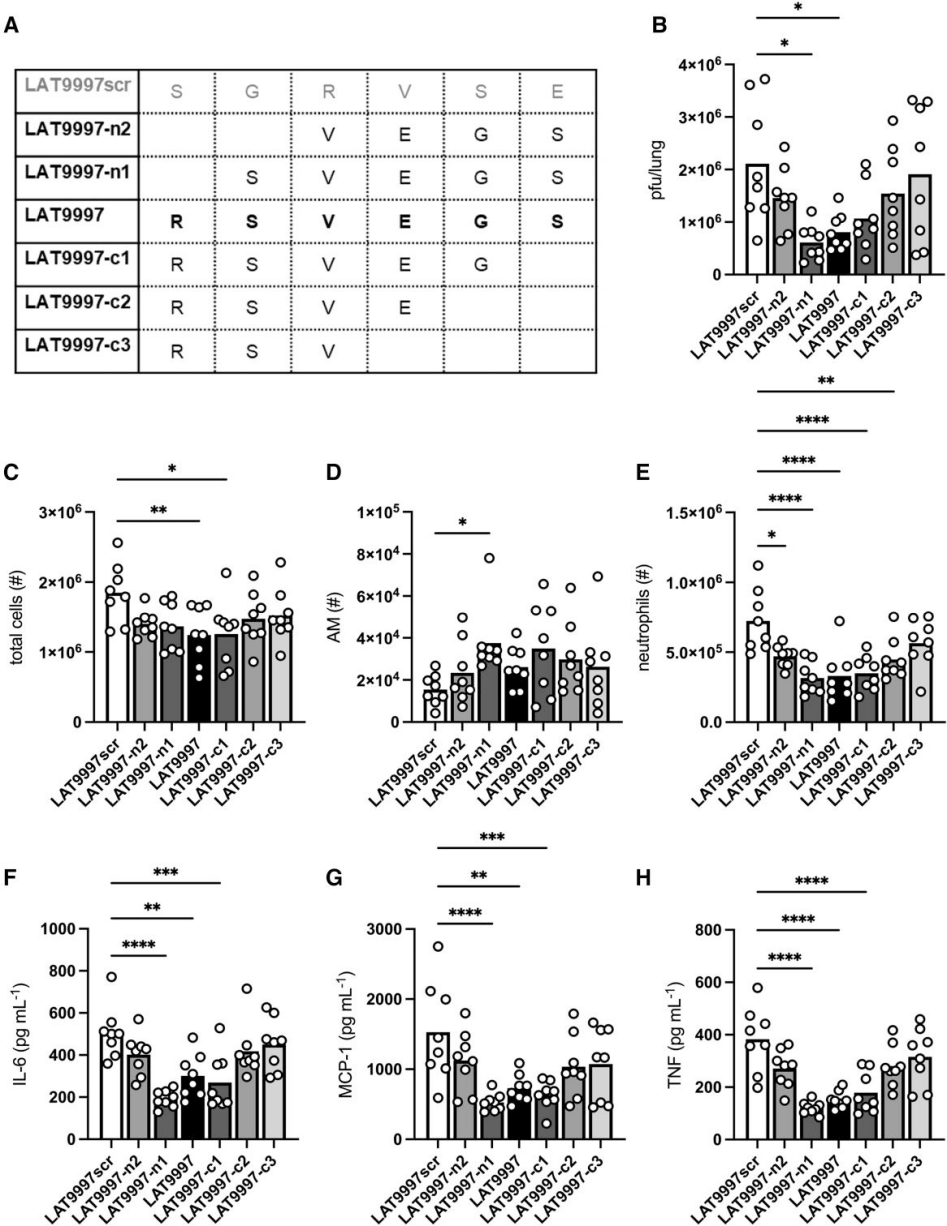
Analysis of the structure-activity relationship of LAT9997 reveals core residues required for activity. Groups of male C57BL/6 mice received daily intranasal treatment with 10 mg·kg^−1^ of LAT9997; the shorter derivatives LAT9997-n1, LAT9997-n2, LAT9997-c1, LAT9997-c2, and LAT9997-c3; or the scrambled control LAT9997scr (schematic in *A*), from 1 day postinfection with 10^4^ plaque-forming units of HKx31 influenza A virus. *B–H*, BAL fluid and lung tissues were collected at 3 days postinfection. *B*, Lung viral loads (plaque-forming units/lung) were measured by a standard plaque assay. *C–E*, Numbers of total cells, alveolar macrophages (AM), and neutrophils in the BAL fluid as determined by flow cytometry. *F–H*, BAL fluid concentrations of IL-6, MCP-1/CCL2, and TNF were determined by cytokine bead array. Data are presented as the mean from a single experiment, with each data point representing an individual animal (n = 8). *P < .05, **P < .01, ***P < .001, ****P < .0001. One-way analysis of variance with Tukey's multiple-comparisons test.

We performed structure-activity relationship (SAR) studies to identify the amino acids of LAT9997 required for therapeutic efficacy. Shorter peptide derivatives of LAT9997 were generated (Figure 4*A*) by sequential removal of 1 or 2 amino acids from the N-terminus (LAT9997-n1 and LAT9997-n2; 5– and 4–amino acid peptides, respectively) or 1, 2, or 3 amino acids from the C-terminus (LAT9997-c1, LAT9997-c2, and LAT9997-c3) as well as a scrambled control 6-mer peptide, LAT9997scr. Infected mice received 10 mg·kg^−1^ of LAT9997 or a derivative from 1 dpi and were compared with a LAT9997scr control cohort.

Lung viral burden (pfu/lung) at 3 dpi was significantly reduced (2.6-fold) by treatment with 10 mg·kg^−1^ of LAT9997. Similar results were seen with peptides lacking the arginine from the N-terminus (LAT9997-n1) or the serine from the C-terminus (LAT9997-c1). However, further trimming of N-terminal (LAT9997-n2) or C-terminal (LAT9997-c2 and LAT9997-c3) residues reduced in vivo activity.

A decrease in total BAL cellularity was observed following treatment with all LAT9997 derivatives when compared with LAT9997scr treatment, significantly so with LAT9997 and LAT9997-c1 (Figure 4*C*). Importantly, AMs increased across all treatment groups; yet, a significant increase was induced only by LAT9997-n1 (Figure 4*D*). Marked reductions in neutrophils were observed following treatment with all LAT9997 derivatives except for LAT9997-c3, with LAT9997 and the 5–amino acid variants LAT9997-n1 and LAT9997-c1 inducing the most profound reductions (Figure 4*E*). Concentrations of key proinflammatory cytokines IL-6, CCL2/MCP-1, and TNF were more than halved upon treatment with LAT9997 and the two 5–amino acid variants LAT9997-n1 and LAT9997-c1, while the effect was lost with the shorter peptide variants (Figure 4*F–H*).

Together, these SAR studies demonstrate that trimming of >1 amino acid from either terminus of LAT9997 abrogated its therapeutic effect during IAV infection. Indeed, it appears that a 4–amino acid core sequence of SVEG is required, as the 3 most potent peptides, LAT9997, LAT9997-n1, and LAT9997-c1, all contain this sequence, whereas the scrambled variant LAT9997scr was inactive.

## DISCUSSION

In addition to the pandemic potential of IAV, seasonal influenza causes substantial global morbidity and mortality, creating significant health care and economic burdens [29]. Approximately 40% of hospitalized patients with influenza are diagnosed with acute pneumonia [30]. While inflammation is critical for the resolution of infection, excessive or uncontrolled inflammation can promote lung injury. The alveolar epithelium provides at least 90% of the resistance to protein transport across the epithelial-endothelial barrier [31]. Epithelial cell injury and death compromise the epithelial barrier layer, leading to leakage of proteinaceous edema fluid into the interstitial and alveolar spaces [17, 18]. Critically, this fluid reduces alveolar gas exchange and can result in respiratory dysfunction, which can progress to acute respiratory distress syndrome [1, 32, 33]. As lung injury largely results from the host's inflammatory response, traditional IAV antiviral therapy alone may not be effective at limiting the development of severe pulmonary disease. Critically, LAT9997 treatment limited hyperinflammation and lung damage with reduced total protein and DAMP levels in BAL fluids (Figure 2). Histologic analysis also revealed reduced epithelial cell damage and death (Figure 2*D*). The release of DAMPs from injured, dead, or dying cells has the potential to amplify inflammation via activation of Toll-like receptors, leading to secretion of cytokines such as TNF, IL-6, and MCP-1. LAT9997 treatment resulted in a broad reduction in local levels of these cytokines (Figure 3), all of which are associated with influenza-induced cytokine storms [19, 20]. Levels of IFNγ were reduced, and TNF and IFNγ can promote epithelial cell death and alter barrier permeability [34, 35]. Thus, in the context of severe influenza, LAT9997 treatment may regulate the escalating cycle of inflammation and lung injury.

IAV infection of epithelial cells and AMs results in the local production of cytokines and chemokines, critical for mediating the rapid infiltration of immune cells, including neutrophils and IMs [13, 16, 36]. Neutrophils have often been considered “double-edged swords,” being associated with infection clearance and immunopathology [37]. In the context of IAV, neutrophils are necessary for protection, with potent in vivo depletion resulting in worse disease resistance and increased extrapulmonary spread in mice [17, 38, 39]. Yet, a dampening of their effects can be beneficial, as antibody-mediated partial depletion of neutrophils ameliorates disease [40]. A correlate of the protection imparted by 20 mg·kg^−1^ of LAT9997 treatment was a significant reduction in, but not ablation of, airway neutrophils (Figure 3*D*). This may reflect decreased infiltration, as LAT9997 significantly reduced concentrations of neutrophil chemoattractants KC/CXCL1 and MCP-1/CCL2 in the airways (Figure 3*F* and 3*K*). Antagonism of CXCL1/2 in vivo has been shown to limit neutrophil infiltration and pulmonary damage [38, 41, 42]. LAT9997 did not alter neutrophil numbers in the blood (Supplementary Figure 4*B*), indicating that reduced neutrophil infiltration was not due to impaired mobilization of mature neutrophils from bone marrow reserves in response to diminished inflammatory cues [43]. Additionally, IL-6 can protect neutrophils from influenza-induced cell death [44]; thus, the contemporaneous significant reduction of local levels of this cytokine (Figures 3*E* and 4*F*) may have influenced neutrophil abundance within the airways. By contrast, no significant change in IM abundance was observed with LAT9997 treatment (Supplementary Figure 3*B*) despite this compound inducing a profound reduction in the level of monocyte/macrophage chemokine MCP-1/CCL2 (Figure 3*F*) [42, 45].

In addition to infection-induced neutrophilia, we observed significant lymphopenia, which is a common early clinical feature of severe IAV infections [26, 46] and a recognized risk factor for hospital-acquired secondary infections [47, 48]. LAT9997 administration was sufficient to significantly lessen the extent of lymphopenia, resulting from the greater abundance of B and T cells, but not natural killer cells, in the blood. This resulted in a significantly lower blood NLR, with increased NLR being a clinically relevant measure of influenza disease severity and prognosis [27, 28]. Indeed, NLR changes could be a candidate pharmacodynamic marker for prospective clinical trials of LAT9997 or future derivatives thereof in the context of influenza.

Of the approved peptides for clinical use, the majority are cyclic, which, when compared with linear peptides, can be more conformationally stable and less susceptible to various peptidases, with better cell membrane permeability and longer half-lives [49, 50]. In our hands, shorter linear peptides were much more soluble than LAT8881. Treatment with the shorter and linear variant LAT9997 induced similar effects to LAT8881 in our influenza model, implying no dramatic difference in the mode of action for these 2 peptides. Yet, relative to LAT8881, a higher molar concentration of LAT9997 was required (1.5–3 times more), potentially reflecting the disadvantages of linear peptides as a drug modality, which would be offset by cheaper and less complex production (ie, fewer steps in the manufacturer process and increased yield) and simpler formulation. In addition to establishing that LAT9997 affords similar protection against IAV, we have further narrowed the active motif to at least a 5–amino acid sequence. Indeed, the positive in vivo effect of LAT9997 on influenza disease was conserved with the removal of a single N- or C-terminal amino acid (Figure 4). Defining the minimal chemically active motif via additional SAR analysis can be used in molecular modeling to select nonpeptidic mimetics that could be more suitable for alternate administration routes, including oral delivery.

We previously reported that the cyclic peptide LAT8881 and its natural metabolite LAT9991F provided a therapeutic benefit in our preclinical IAV infection model [13]. We have now demonstrated that LAT9997—a linear 6–amino acid sequence within the cyclic region of LAT8881 and LAT9991F—reproduces our previous findings and is sufficient to significantly ameliorate severe influenza infection. Importantly, LAT9997 can significantly moderate potentially deleterious neutrophil infiltration to the site of infection, which was not observed by treatment with LAT8881. Additionally, valuable SAR data obtained by using modified LAT9997 variants will help to inform future clinical development. In sum, like the cyclic peptides LAT8881 and LAT9991F, the linear LAT9997 peptide is a potential host-directed novel therapeutic for severe IAV infection that could be used in combination with approved influenza antivirals.

## Supplementary Data

Supplementary materials are available at *The Journal of Infectious Diseases* online (http://jid.oxfordjournals.org/). Supplementary materials consist of data provided by the author that are published to benefit the reader. The posted materials are not copyedited. The contents of all supplementary data are the sole responsibility of the authors. Questions or messages regarding errors should be addressed to the author.

## Supplementary Material

jiad566_Supplementary_Data

## Notes

***Acknowledgments*.** We acknowledge Monash FlowCore at the Monash Health Translation Precinct for the provision of equipment, advice, and support used in this study. We also acknowledge the services of the Monash Histology Platform at the Monash Health Translation Precinct.

***Author contributions.*** Conceived and designed experiments: C. M. H., A. C. W., L. K. E., A. J. G., and M. D. T. Performed experiments: M. D. T., C. M. H., A. C. W., M. A. L., and C. H. Analyzed data: M. D. T., C. M. H., M. A. L., M. L., and A. C. W. Wrote the manuscript: A. C. W., C. M. H., A. J. G., L. K. E., and M. D. T.

***Data availability statement.*** The data that support the findings of this study are available from the corresponding author upon reasonable request.

***Financial support*.** This work was supported by the Victorian State Government Operational Infrastructure Scheme; Lateral Pharma Pty Ltd; and the Victorian Medical Research Acceleration Fund. Funding to pay the Open Access publication charges for this article was provided by Lateral Pharma Pty Ltd.
