# Supplementary material for: Harnessing Endogenous Peptide Compounds as Potential Therapeutics for Severe Influenza
Source: J Infect Dis. 2023 Dec 7;230(2):e384–94. doi: 10.1093/infdis/jiad566 (PMC11326819; doi:10.1093/infdis/jiad566)
Supplement: jiad566_Supplementary_Data [file jiad566_supplementary_data.pdf]

SUPPLEMENTAL METHODS:

Influenza virus infection of mice

Mice were weighed daily and assessed for clinical signs of disease on a scale of 0 to 3 (0 = no visible signs; 1 = slight ruffling of fur; 2 = ruffled fur, reduced mobility; 3 = ruffled fur, reduced mobility, rapid breathing). Animals that lost 20% of their original body weight or displayed severe clinical signs of disease (a score of 3) were euthanised.

Flow cytometry on BAL and blood cells

Cells in the BAL fluid were isolated by centrifugation and treated with red blood cell lysis buffer (Sigma Aldrich, Cat# R7757) for 5 min. The reaction was quenched by washing the cells in FACS buffer (PBS containing 2% (v/v) FBS and 2 mM EDTA). BAL cells were then incubated with fluorescently labelled antibodies at 4°C for 20 mins in FACS buffer and in the presence of Fc receptor blocking monoclonal antibody against CD16/CD32 (clone 93, Thermo Fisher Scientific, Cat# 139311, RRID:AB_468898) to limit non-specific antibody binding. BAL cells were stained in FACS buffer with monoclonal antibodies to Siglec-F (clone E50-2440, BD Biosciences, Cat# 565527, RRID:AB_2732831), NK1.1 (clone PK136, BioLegend, Cat# 108727, RRID:AB_2132706), CD3ε (clone 145-2C11, BioLegend, Cat# 100355, RRID:AB_2565969), CD11c (clone HL3, BD Biosciences, Cat# 564080, RRID:AB_2738580), CD64 (clone X54-5/7.1, BioLegend, Cat# 139311, RRID:AB_2563846), Ly6C (clone AL-21, BD Biosciences, Cat# 562727, RRID:AB_2737748), Ly6G (clone 1A8, Cat# 551461, RRID:AB_394208, BD Biosciences), and I-A^{b} (clone AF6-120.1, BD Biosciences, Cat# 562823, RRID:AB_2737818) and the Zombie Aqua viability dye (Cat# 423102; BioLegend). Total live cells (Zombie Aqua viability dye^{-}), neutrophils (Ly6G^{+} Ly6C^{int}), NK cells (NK1.1^{+} CD3^{-}), T cells (NK1.1^{-} CD3^{+}), IM

(CD64$^+$ Ly6G$^-$ Ly6C$^+$), AM (CD64$^+$ CD11c$^+$ Siglec-F$^+$) and DCs (CD11c$^+$ I-A^{b+}) were quantified by flow cytometry using a BD LSRFortessa™ X-20 (BD Biosciences, RRID:SCR_019600) or Aurora flow cytometer (Cytek Biosciences, RRID:SCR_019826) and FlowJo™ 10 analysis software (BD Biosciences, RRID:SCR_008520). Cells were enumerated using a standard amount of blank calibration particles (ProSciTech) as determined using a haemocytometer.

In the indicated experiments, blood was obtained directly from the heart and stored in K3 EDTA-containing blood collected tubes (Sarstedt). An equal volume of blood was then treated with red blood cell lysis buffer (Sigma Aldrich) for 5 min. The reaction was quenched by washing the cells in FACS buffer (PBS containing 2% (v/v) FBS and 2 mM EDTA). Blood cells were then incubated with fluorescently labelled antibodies at 4°C for 20 min in FACS buffer and in the presence of Fc receptor blocking monoclonal antibody against CD16/CD32 (clone 93, Thermo Fisher Scientific, Cat# 139311, RRID:AB_468898) to limit non-specific antibody binding. BAL cells were stained in FACS buffer with monoclonal antibodies to CD45 (clone 30-F11, BioLegend, Cat# 103138, RRID:AB_2563061), NK1.1 (clone PK136, BioLegend, Cat# 108727, RRID:AB_2132706), CD3ε (clone 145-2C11, BioLegend, Cat# 100355, RRID:AB_2565969), CD4 (clone RM4-5, BD Biosciences, Cat# 558107, RRID:AB_397030), CD8a (clone 53-6.7, Cat# 100762, BioLegend, RRID:AB_2564027), Ly6C (clone AL-21, BD Biosciences, Cat# 562727, RRID:AB_2737748), Ly6G (clone 1A8, Cat# 551461, BD Biosciences, RRID:AB_394208), B220 (clone RA3-6B2, BioLegend, Cat# 103228, RRID:AB_492874), I-A^b (clone AF6-120.1, BD Biosciences, Cat# 562823, RRID:AB_2737818) and the Zombie NIR viability dye (Cat# 423105, BioLegend). Viable (Zombie NIR viability dye$^-$) total blood leukocytes (CD45$^+$), neutrophils (Ly6G$^+$ Ly6C^{int}), B cells (B220$^+$ I-A^{b+} CD3$^-$), T cells (CD3$^+$), CD8$^+$ T cells (CD3$^+$ CD8$^+$), CD4$^+$ T cells (CD3$^+$ CD4$^+$), double negative T cells (CD3$^+$ CD4$^-$ CD8$^-$), NK cells (NK1.1$^+$ CD3$^-$) and monocytes

($Ly6C^{hi}$ $I\text{-}A^{b+}$), were quantified by flow cytometry using an Aurora flow cytometer (Cytek Biosciences, RRID:SCR_019826) and FlowJo™ 10 analysis software (BD Biosciences, RRID:SCR_008520). Cells were enumerated per mL of original blood using a standard amount of blank calibration particles (ProSciTech, Cat# QBCP-60-5) as determined using a haemocytometer.

Assessment of lung damage

LDH and ATP levels in BAL fluid supernatants were used as indicators of lung damage [13]. Levels of LDH in BAL fluid supernatant were determined using a CytoTox 96 Non-radioactive Cytotoxicity Assay (Promega), according to the manufacturer's instructions. Levels of ATP in BAL fluid supernatant were determined using a CellTiter-Glo 2.0 Cell Viability Assay (Promega), according to the manufacturer's instructions. Levels of protein in BAL fluid supernatant were determined using a Pierce™ BCA Protein Assay Kit (Thermo Fisher Scientific). Levels of S100A10 in BAL fluid were determined using ELISA (Cloud-Clone Corporation), according to the manufacturer's instructions.

Histological assessment of lung immunopathology

In the indicated experiments, mice were sacrificed via intraperitoneal injection of sodium pentobarbital, and their lungs were immediately inflated and fixed in 10% formalin for at least 24 h and then processed in paraffin wax. Longitudinal tissue sections (4 μm) were prepared and stained with hematoxylin and eosin (H&E). Tissues were graded for alveolitis and peribronchial inflammation on a subjective scale of 0-5 (0 = no inflammation, 1 = very mild, 2 = mild, 3 = moderate, 4 = marked, and 5 = severe inflammation), as previously described [13]. Sections were also scored for features of epithelial damage such as the presence of debris in the airspace, epithelial denudation, and thickening of the epithelial wall (0 = no obvious

damage, 1 = mild, 2 = moderate, 3 = marked, and 4 = severe). Sections were blinded and randomised, and samples corresponding to the least severe and most severe were assigned scores of 0 and 4/5, respectively. All other samples were graded in 5 random fields by three independent readers. Lung sections were viewed on an Olympus BX60 microscope and photographed at ×10 magnification with an Olympus DP74 colour camera running from Olympus cellSens Dimension software.

Immunohistochemical staining of lung tissue sections for cell death

Terminal deoxynucleotidyl transferase-mediated dUDP nick-end labelling (TUNEL) assay was performed on lung tissue sections (4 µm) using the ApopTag Peroxidase *In Situ* Apoptosis Detection Kit (Merck Millipore), according to the manufacturer's instructions. Sections were counterstained with hematoxylin. Lung sections were viewed on an Olympus BX60 microscope (Olympus) and photographed at 10x magnification with an Olympus DP74 colour camera using Olympus cellSens Dimension software. TUNEL staining intensity (% positive pixel intensity per field of view) was quantified using ImageJ software. Colour deconvolution was performed, and a threshold was set on diaminobenzidine (DAB) intensity, with the same parameters applied to all sections. Five random fields per section were analysed.

Data and Statistical Analysis

Data were tested for normality and analysed by GraphPad Prism version 9 software (Graphstats Technologies). When comparing three or more sets of values, a one-way analysis of variance (ANOVA) was used with either a Tukey's or Dunnett's (when comparisons were only made to IAV-infected PBS control group) multiple comparisons post-hoc test. A Student's *t*-test was used when comparing 2 values (two-tailed, two-sample equal variance). Survival proportions

were compared using the Mantel-Cox log-rank test. A *P*-value < 0.05 was considered statistically significant.

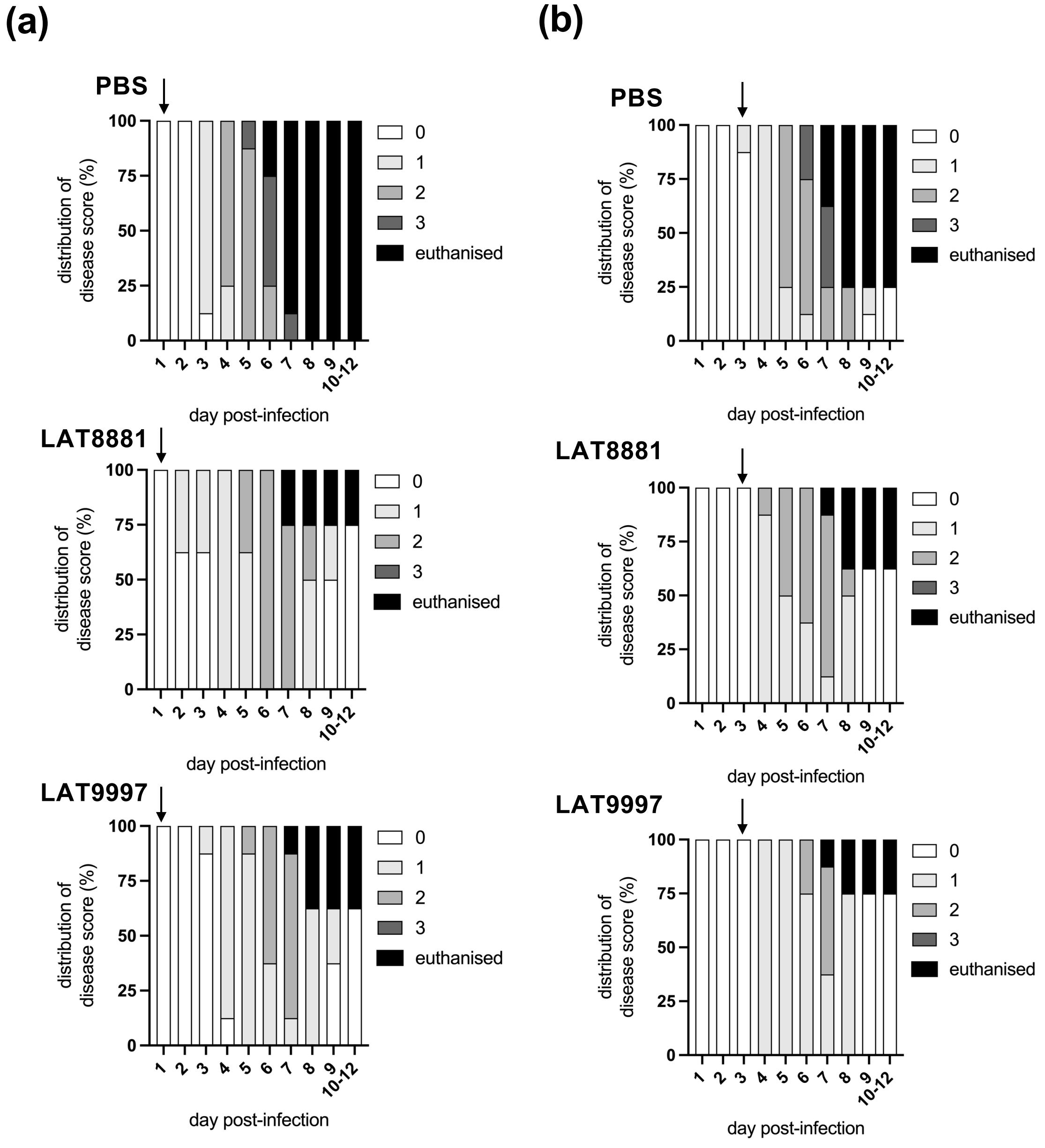

Supplementary figure 1. Delayed treatment with LAT8881 and LAT9997 improves resistance to IAV infection *in vivo*. Groups of male C57BL/6 mice (n = 8) received daily i.n. treatment with 20 mg kg^{-1} of LAT8881, PBS (vehicle control), and 20 mg kg^{-1} of LAT9997 from **(a)** 1 day post-infection or **(b)** 3 days post-infection with 10^4 pfu of HKx31 IAV. The proportion of mice (%) in the treatment groups that were assigned each score of clinical disease (scale 0-3 and euthanised) is shown for all days monitored post-infection. The downward arrows indicate the day treatment was initiated.

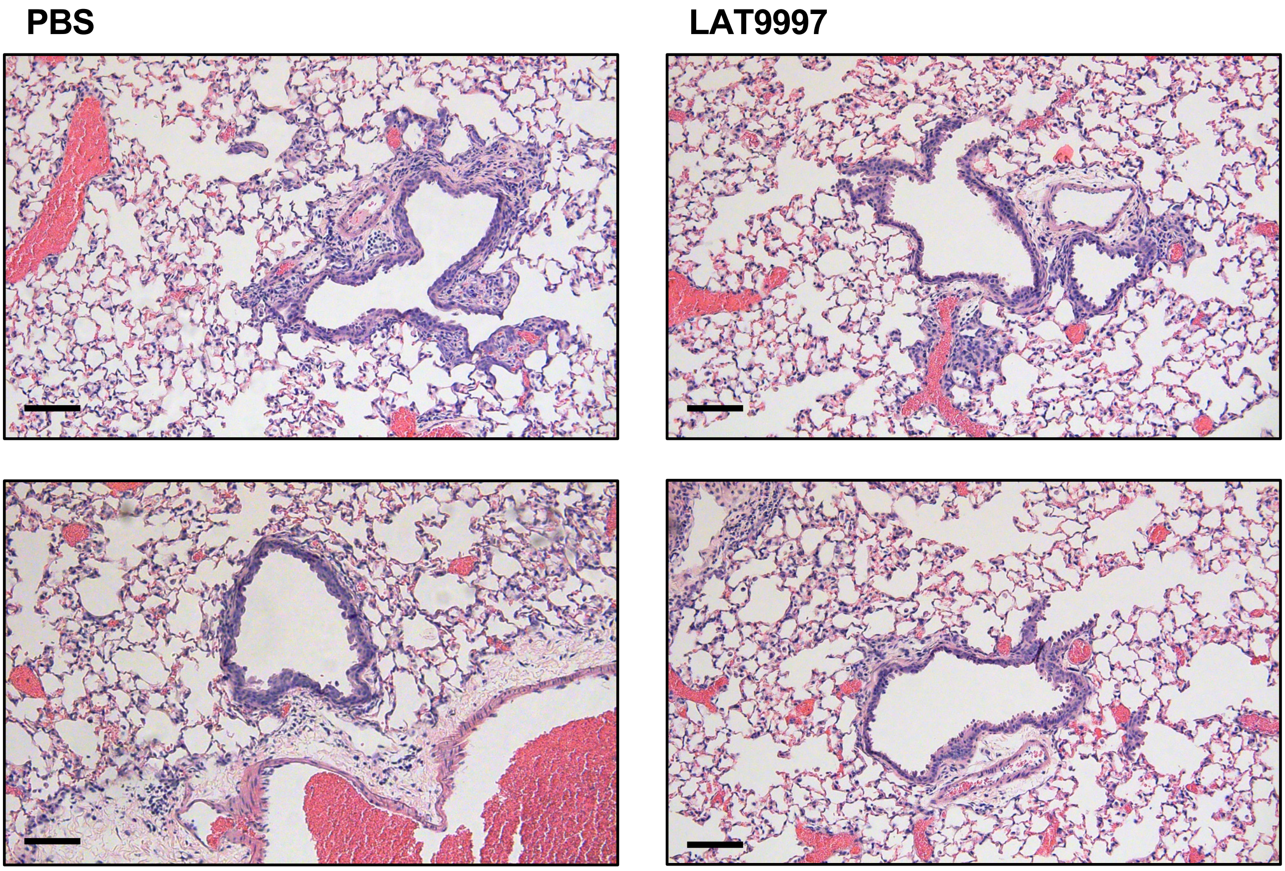

Supplementary figure 2. LAT9997 treatment of severe IAV infection aids resolution of pulmonary immunopathology. Groups of male C57BL/6 mice received daily i.n. treatment with 20 mg kg^{-1} of LAT9997 daily from 3 days post-infection with 10^4 pfu of HKx31 IAV until 10 days post-infection. IAV-infected control mice received PBS alone. Histological analysis of H&E-stained lung tissue sections at 12 days post-infection was performed, and representative images from two mice per treatment group at 10x magnification (scale bar = 100 µm) are shown.

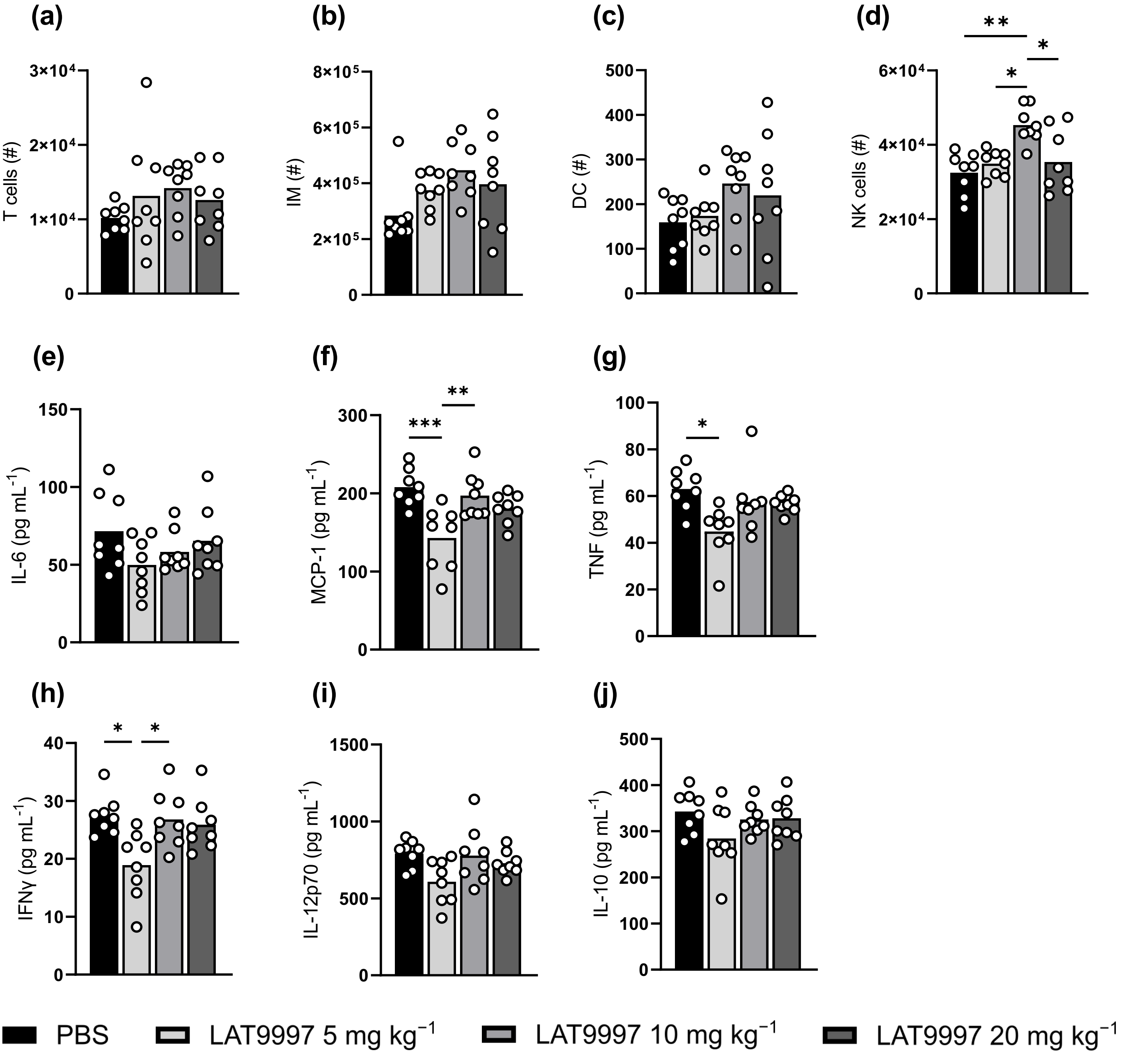

Supplementary figure 3. LAT9997 treatment of mice with severe IAV infection does not alter total cell numbers, T cells, inflammatory macrophages (IM) or dendritic cells (DC) in the BAL fluid, nor serum cytokines. Groups of male C57BL/6 mice received daily i.n. treatment with 5, 10, or 20 mg kg^{-1} of LAT9997 from 1 day post-infection with 10^4 pfu of HKx31 IAV. IAV-infected control mice received PBS alone. BAL fluid, lung tissues, and serum were collected at 3 days post-infection. Numbers (#) of **(a)** T cells, **(b)** inflammatory macrophages (IM), **(c)** dendritic cells (DC), and **(d)** natural killer (NK) cells in the BAL fluid as determined by flow cytometry. Data are presented as the mean from a single experiment, with each data point representing an individual animal. $n = 8$ per group. *$P < 0.05$, **$P < 0.01$, one-way ANOVA with Tukey's multiple comparisons test. Serum concentrations of **(e)** IL-6, **(f)** MCP-1, **(g)** TNF, **(h)** IFNγ, **(i)** IL-12p70, and **(j)** IL-10 were determined by cytokine bead array. Data are presented as the mean from a single experiment, with each data point representing an individual animal. $n = 8$ per group. *$P < 0.05$, **$P < 0.01$, ***$P < 0.001$, one-way ANOVA with Tukey's multiple comparisons test.

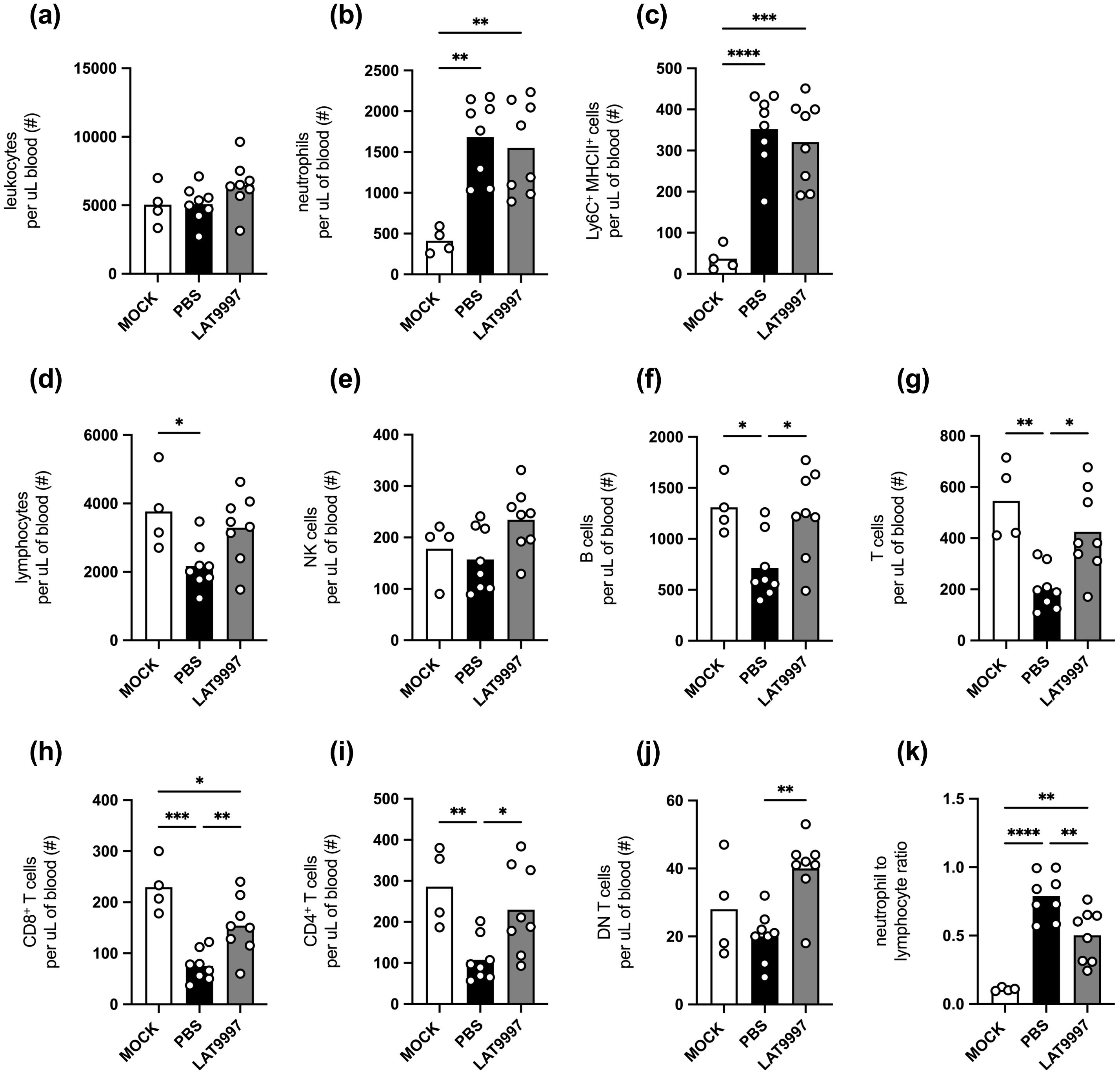

Supplementary figure 4. LAT9997 treatment of mice with severe IAV infection reduces blood lymphopenia and the neutrophil to lymphocyte ratio. Groups of male C57BL/6 mice received daily i.n. treatment with 20 mg kg^{-1} LAT9997 from 1 day post-infection with 10^4 pfu of HKx31 IAV. MOCK-infected and IAV-infected control mice received PBS alone. Blood was collected at 3 days post-infection. Numbers (#) per µl of blood of **(a)** leukocytes, **(b)** neutrophils, **(c)** $Ly6C^+$ $MHCII^+$ monocytes, **(d)** lymphocytes, **(e)** natural killer (NK) cells, **(f)** B cells, **(g)** T cells, **(h)** $CD8^+$ T cells, **(i)** $CD4^+$ T cells, and **(j)** double negative (DN) T cells in the BAL fluid as determined by flow cytometry. Neutrophil to lymphocyte ratio **(k)** was calculated. Data are presented as the mean from a single experiment, with each data point representing an individual animal. $n = 4\text{-}8$ per group. $*P < 0.05$, $**P < 0.01$, $***P < 0.001$, $****P < 0.0001$, one-way ANOVA with Tukey's multiple comparisons test.
